# Unveiling the Arsenal of Apple Bitter Rot Fungi: Comparative Genomics Identifies Candidate Effectors, CAZymes, and Biosynthetic Gene Clusters in *Colletotrichum* Species

**DOI:** 10.3390/jof10070493

**Published:** 2024-07-16

**Authors:** Fatemeh Khodadadi, Dianiris Luciano-Rosario, Christopher Gottschalk, Wayne M. Jurick, Srđan G. Aćimović

**Affiliations:** 1 Department of Plant Pathology and Microbiology, University of California, Riverside, Riverside, CA 92521, USA; fatemehk@ucr.edu; 2 Food Quality Laboratory, U.S. Department of Agriculture, Agriculture Research Service, Beltsville Agricultural Research Center, Beltsville, MD 20705, USA; d.lucianorosario@usda.gov (D.L.-R.);; 3 Appalachian Fruit Research Station, U.S. Department of Agriculture, Agriculture Research Service, Kearneysville, WV 25430, USA; christopher.gottschalk@usda.gov; 4 Alson H. Smith Jr. Agricultural Research and Extension Center, School of Plant and Environmental Sciences, Virginia Polytechnic Institute and State University, Winchester, VA 22602, USA

**Keywords:** secondary metabolites, CAZymes, *Colletotrichum* spp., bitter rot, apple, effectors

## Abstract

The bitter rot of apple is caused by *Colletotrichum* spp. and is a serious pre-harvest disease that can manifest in postharvest losses on harvested fruit. In this study, we obtained genome sequences from four different species, *C*. *chrysophilum*, *C. noveboracense*, *C. nupharicola*, and *C. fioriniae*, that infect apple and cause diseases on other fruits, vegetables, and flowers. Our genomic data were obtained from isolates/species that have not yet been sequenced and represent geographic-specific regions. Genome sequencing allowed for the construction of phylogenetic trees, which corroborated the overall concordance observed in prior MLST studies. Bioinformatic pipelines were used to discover CAZyme, effector, and secondary metabolic (SM) gene clusters in all nine *Colletotrichum* isolates. We found redundancy and a high level of similarity across species regarding CAZyme classes and predicted cytoplastic and apoplastic effectors. SM gene clusters displayed the most diversity in type and the most common cluster was one that encodes genes involved in the production of alternapyrone. Our study provides a solid platform to identify targets for functional studies that underpin pathogenicity, virulence, and/or quiescence that can be targeted for the development of new control strategies. With these new genomics resources, exploration via omics-based technologies using these isolates will help ascertain the biological underpinnings of their widespread success and observed geographic dominance in specific areas throughout the country.

## 1. Introduction

More than 200 species of the fungal genus *Colletotrichum* devastatingly affect a wide array of agricultural crops including vegetables, fruits, forest trees, cereals, and legumes by causing anthracnose, fruit rot, and blights [1,2,3]. These fungi are ranked among the top ten most significant pathogens in the world because of their omnipresence and economic impact, and they provide model pathosystems for plant–pathogen interaction studies [4]. Unlike some *Colletotrichum* species with narrow host ranges [5], the *C. acutatum* species complex (CASC) and the *C. gloeosporioides* species complex (CGSC) exhibit broader host specificity. These complexes encompass multiple species that cause rot disease in apple and various other crops, including small fruits, citrus, and peach. Some of the dominant species causing apple bitter rot in the Mid-Atlantic are *C. fioriniae*, *C. chrysophilum,* and *C. noveboracense* [6,7]. The enormous economic loss imposed by bitter rot on the apple industry could be managed by fungicide application or using resistant cultivars, which are currently not available. However, these strategies are unattainable due to the diverse genetic makeup of *Colletotrichum* species, lack of known genetic resistance within domesticated apple germplasm [8,9], and the development of fungicide-resistant strains. Therefore, a thorough investigation at the genomic level to explore and discover potential pathogenic mechanisms of *Colletotrichum* species is needed.

Hemibiotrophism is the most common lifestyle utilized by *Colletotrichum* species. To carry out this lifestyle, *Colletotrichum* spp. use various specialized structures to colonize multiple plant parts such as leaves, stems, and fruits. Upon landing on the plant tissue, fungal conidia adhere to the host surface and form specialized structures called appressoria which allow the fungus to penetrate the host cuticle via a penetration peg forming at the base of appressorium [10,11]. The primary hyphae emerging from the penetration peg accelerate nutrient acquisition from colonized plant tissues. The switch from biotrophy to the necrotrophic phase is directed by the production of secondary infectious hyphae which then will invade neighboring cells and kill the host tissue [12,13].

Establishing a successful infection by *Colletotrichum* species relies on a large spectrum of expressed and secreted pathogenicity and virulence factors such as effector proteins, carbohydrate active enzymes (CAZyme), and secondary metabolites [14]. Effectors are both proteins or non-proteinaceous molecules secreted into plant hosts to facilitate infection, compromise the plant defense system, and/or in some cases trigger the host defense responses [10]. Given the diverse host range of *Colletotrichum* spp., there are specialized effectors developed for fine-tuned adjustments to specific hosts or conserved effectors mediating infection processes for multiple hosts [15]. CAZymes directly contribute to the degradation of plant cell wall components to liberate carbohydrates upon host colonization that also serve as carbon sources for fungal growth and proliferation [16,17]. CAZymes are categorized into six major groups: glycoside hydrolases (GHs), glycosyl transferases, polysaccharide lyases (PLs), carbohydrate esterases (CEs), auxiliary activity enzymes (AA), and carbohydrate-binding modules (CBMs) [18]. CAZymes in *Colletotrichum* spp. outnumber those of any other ascomycete fungi sequenced so far [19]. Fungi owe their adaptability, survival, and pathogenicity to secondary metabolites (SMs), which in a fungal context, refers to the production of molecules termed natural products or secondary metabolites that are not part of primary metabolism. SMs are small molecules, synthesized by enzymes encoded by biosynthetic gene clusters (BGCs) in their genomes [20,21]. Secondary metabolites are of great research interest as these molecules include toxins and beneficial natural products such as antibiotics, and they are important for ecological interactions of fungi with other microbes and their environment. In comparison to fungi like *Verticillium* and *Fusarium*, some *Colletotrichum* species contain higher numbers of SM gene clusters in genomes varying from 41 in *C*. *salicis* to 73 in *C. truncatum* [22]. The identification and comparison of pathogenicity gene repertoires of different *Colletotrichum* species is paramount to understand plant–pathogen interaction, assess the present risks of these species, and ultimately develop economic and effective disease management approaches.

Comparative genomics and genome-scale analyses of *Colletotrichum* spp. offer an invaluable platform for unraveling the intricate patterns underlying speciation, pathogenesis, host specificity, and evolutionary relationships within this genus [23,24]. Thanks to advancements in high-throughput sequencing, over 250 fully sequenced genomes of *Colletotrichum* are currently available in the National Center for Biotechnology Information (NCBI) repository [14]. However, given the growing number of species and species complexes in the genus, more studies on genome sequencing and genome-mining analyses are needed to fill the existing research gaps. Therefore, conducting comparative genomics analyses and an analysis of the pathogenicity of gene repertoires of *Colletotrichum* species in CASC and CGSC, particularly for newly identified species like *C. noveboracense* and *C. chrysophilum* in apple, not only will shed light on host specificity, the pathogenic adaptation of these species, and evolutionary relationships but will also help to evaluate the present and future risk of these pathogens in the agricultural industry.

The aim of the current study was to utilize genomics in different *Colletotrichum* spp. to: 1. provide genomics resources for a total of nine isolates of four *Colletotrichum* species (*C. fioriniae*, *C. chrysophilum*, *C. noveboracense*, and *C. nupharicola*) that have not yet been sequenced and/or are from isolates from a specific geographic region, 2. construct phylogenetic trees using newly sequenced genome data to compare to previous MLST findings, 3. discover and discuss CAZyme, effector, and SM gene clusters within and across *Colletotrichum* spp. Our immediate and long-term goals are to identify targets for functional gene studies that may mediate pathogenicity, virulence, and/or quiescence in the different fungal species that can be targeted for the development of new control strategies (e.g., dsRNA development). In addition, we would like to explore more omics-based technologies (e.g., transcriptomics, proteomics, and metabolomics) using these isolates and their genomes to ascertain the biological underpinnings of their widespread success and observed geographic dominance in specific areas throughout the country.

## 2. Materials and Methods

### 2.1. Fungal Strains, Genomic DNA Extraction, and Whole Genome Sequencing

Full genome sequences of two isolates of *C. fioriniae* (ACFK5 and ACFK16), three isolates of *C. chrysophilum* (AFK154, AFK26, and PMKnsl-1), and one isolate of *C. noveboracense* (PMBrms-1) were obtained from distinctive lesions on bitter rot-infected apple fruit in New York, Pennsylvania, and Virginia [6]. In addition, the genomes of C. *noveboracense* Coll940 collected from *Juglans nigra* in Oklahoma and two isolates of *C. nupharicola* (CBS470 and Coll922) were isolated from yellow waterlily (*Nuphar lutea*) in Washington and New Jersey, respectively [25]. The genomic DNA was extracted from 5-day-old single-spore cultures via the protocol described by Yelton et al. [26] with slight modification. The whole genome sequencing was performed by the Beijing Genomics Institute (BGI) in Shenzhen, China, using the DNBSEQ short read platform (MGI Tech Co., Ltd. a subsidiary of BGI Group, Shenzhen, China) for 350 bp libraries with the paired-end 150 bp sequencing strategy, as described in the DNBseq De Novo service overview manual (BGI) yielding 46× to 103× coverage, sufficient for genome assembly. Genomes were assembled using SPADES 3.15.2 (Center for Algorithmic Biotechnology, St. Petersburg State University, St. Petersburg, Russia) for isolates Coll940 and AFK154 and MEGAHIT v1.2.9 (Computational Genomics Lab, Hong Kong University of Science and Technology, Kowloon, Hong Kong, China) for isolates CBS470, ACFK5, ACFK16, PMKnsl-1, AFK26, PMBrms-1, and Coll922, and annotated using the MAKER pipeline (University of Utah, Salt Lake City, UT, USA). More detailed materials and methods are described in Khodadadi et al. [27].

### 2.2. Phylogenomics

Three independent approaches were utilized to analyze the phylogeny of the *Colletotrichum* species selected for this study. First, high-throughput average nucleic identity (ANI) was performed through the fastANI software v1.33 (Computational Biology Group, Indian Institute of Science, Bangalore, India; accessed from 9 January 2024 to 1 July 2024) [28]. Here, genome sequences retrieved from NCBI for each of the nine subject genomes were used as input into fastANI with the assemblies for Coll940 and PMBrms-1 (*C. noveboracense*) used as the query list and the other seven assemblies as the reference list. A cluster map of the fastANI results was generated using the ANIclustermap script (https://github.com/moshi4/ANIclustermap; v1.2, accessed from 9 January 2024 to 1 July 2024). The second approach was to use the OrthoFinder (https://github.com/davidemms/OrthoFinder (accessed from 9 January 2024 to 1 July 2024)) [29] to construct a phylogenetic tree based on conserved gene sequence similarity. The protein FASTAs for each of the nine assemblies were used as input with the -m MSA option enabled for the generation of maximum likelihood trees from multiple sequence alignments (MSAs). The resulting newick tree was visualized using the ETE 3 toolkit’s newick tree viewer (Centre for Genomic Regulation, Barcelona, Spain) [30]. Lastly, a tree was generated based on highly conserved single-copy orthologous genes. BUSCO (EMBL Bioinformatics Core Facility, Heidelberg, Germany) was implemented [31,32] to identify single-copy orthologs in each genome. BUSCO was executed in the -m genome mode with the genome sequence FASTA for the ten assemblies. The Glomerellales ODB10 database of BUSCO was implemented as the lineage parameter option. The BUSCO result plot was generated using the generate_plot.py script included with BUSCO. Phylogenetic relationships between the single-copy ortholog genes present in each assembly were investigated using the BUSCO Phylogenomics python pipelines (https://github.com/jamiemcg/BUSCO_phylogenomics; accessed on 5 November 2022). Within this pipeline, MSA was conducted using MUSCLE (Robert C. Edgar, Harvard University, Cambridge) [33] and alignment trimming was performed by trimAl (Salvador Capella-Gutiérrez, Toni Gabaldón, and José M. Sánchez-Pulido, Barcelona, Spain) [34]. A consensus phylogenetic tree was determined using IQ-tree (Alexandros Stamatakis, Heidelberg Institute for Theoretical Studies and Karlsruhe Institute of Technology, Heidelberg, Germany) [34] on the resulting supermatrix generated by the BUSCO_phylogenomics.py pipeline with the -m MFP option enabled and -B for bootstrapping set to 1000. The resulting newick tree was visualized using the ETE 3 toolkit’s newick tree viewer [30]. To complement the phylogenetic analyses conducted using the nine subject genomes, we reperformed the BUSCO phylogeny and fastANI analyses using an additional nine genomes (Supplemental Table S1). These nine genomes were previously identified as belonging to the *acutatum* phylogenetic subclade and offered an opportunity to assess broader species-level relationships with our subject genomes [35]. One species listed in Liu et al. [35] in that subclade, *C. godetiae* (NCBI accession GCA_001663355.1), was not included due to a BUSCO complete score of <90%, suggesting an incomplete genome assembly. Furthermore, AFKH109 has a similarly low BUSCO score and thus was removed from the BUSCO phylogenetic analysis of the 18 assemblies. However, both assemblies were included in the broader fastANI analysis.

### 2.3. Mining for CAZymes, Secondary Metabolic Gene Clusters, and Effectors

Fungal versions of the antibiotics and Secondary Metabolites Analysis SHell (antiSMASH) 6.0 pipeline https://fungismash.secondarymetabolites.org/ (University of Göttingen, Germany) [36] with “relaxed” detection strictness was used for mining our *Colletotrichum* genomes for secondary/specialized metabolite (SM) biosynthetic gene clusters. We used the dbCAN2 meta server for analyzing and predicting six major classes of carbohydrate-active enzymes in our newly sequenced genomes of *Colletotrichum* species (University of Alberta, Edmonton, Canada). HMMER (Sean Eddy, Howard Hughes Medical Institute, Janelia Research Campus, Ashburn, VA, USA) with E-Value < 1e-15, coverage > 0.35, eCAMI (University of Göttingen, Germany) with important_k_mer_number >= 5, k_mer size = 8, and DIAMOND (Benjamin Buchfink and collaborators, Max Planck Institute for Developmental Biology, Tübingen, Germany) with a cut-off E-value of <1e-102 for CAzymes prediction were used as the default thresholds [37]. Candidates found by at least two predictive tools were retrieved. For each CAZy class, the number of enzyme modules and the families they belong to are reported. Finally, we applied EffectorP 3.0 (University of Copenhagen, Denmark) (fungal mode parameter) and SignalP 6.0 mode Eukarya (Technical University of Denmark, Kongens Lyngby, Denmark) with a significance threshold = 0.7 to predict known apoplastic and cytoplasmic effectors in 9 isolates of *Colletotrichum* species [38,39].

## 3. Results and Discussion

### 3.1. Colletotrichum spp. Genomes

As described in Khodadadi et al. [27], a total of 16,621 protein coding genes were predicted in each *Colletotrichum* species assembly. While the genome assembly metrics varied for each of the *Colletotrichum* species sequenced, all genomes had over 95% coverage of complete BUSCOs. The GC content of the genomes sequenced ranged from 49.33 to 53.94%, with *C. fioriniae* and *C. chrysophilum* having the upper and lower ranges, respectively. The estimated genome sizes for the sequenced isolates ranged from 49.33 to 59.08 Mb. The estimated sequencing coverage ranged from 46× to 103×, with the lowest and highest coverages obtained for the *C. nupharicola* and *C. fioriniae* isolates, respectively (Table 1).

### 3.2. Phylogenomic Analysis

A novel *Colletotrichum* species, designated *C. noveboracense*, was identified as the causal agent of apple bitter rot disease in New York and Pennsylvania [6]. Several isolates (PMBrms-1 and AFKH109) from these affected regions, along with a single endophytic isolate from *Juglans nigra* in Oklahoma (Coll940) [25], formed a highly supported distinct clade based on multi-locus sequence analysis (MLSA). Initial phylogenetic analyses using Bayesian inference with a three-gene approach (*ITS*, *TUB2*, and *ApMat*) revealed a well-supported clade (BI PP = 1.0) containing the isolates subsequently identified as *C. noveboracense* [6]. Further Bayesian analysis employing seven loci (*ACT*, *TUB2*, *CAL*, *GAPDH*, *GS*, *ITS*, and *ApMat*) and other locus combinations consistently placed *C. noveboracense* as a sister taxon to *C. nupharicola* (PP = 0.95) within the *Colletotrichum gloeosporioides* species complex (CGSC) [6]. However, *C. nupharicola* is readily distinguishable within CGSC based on morphological characteristics. This host-specific species exhibits significantly slower growth on potato dextrose agar (PDA) and possesses conidia with both length and width exceeding those of other species in CGSC [25,40].

**Figure 1:**
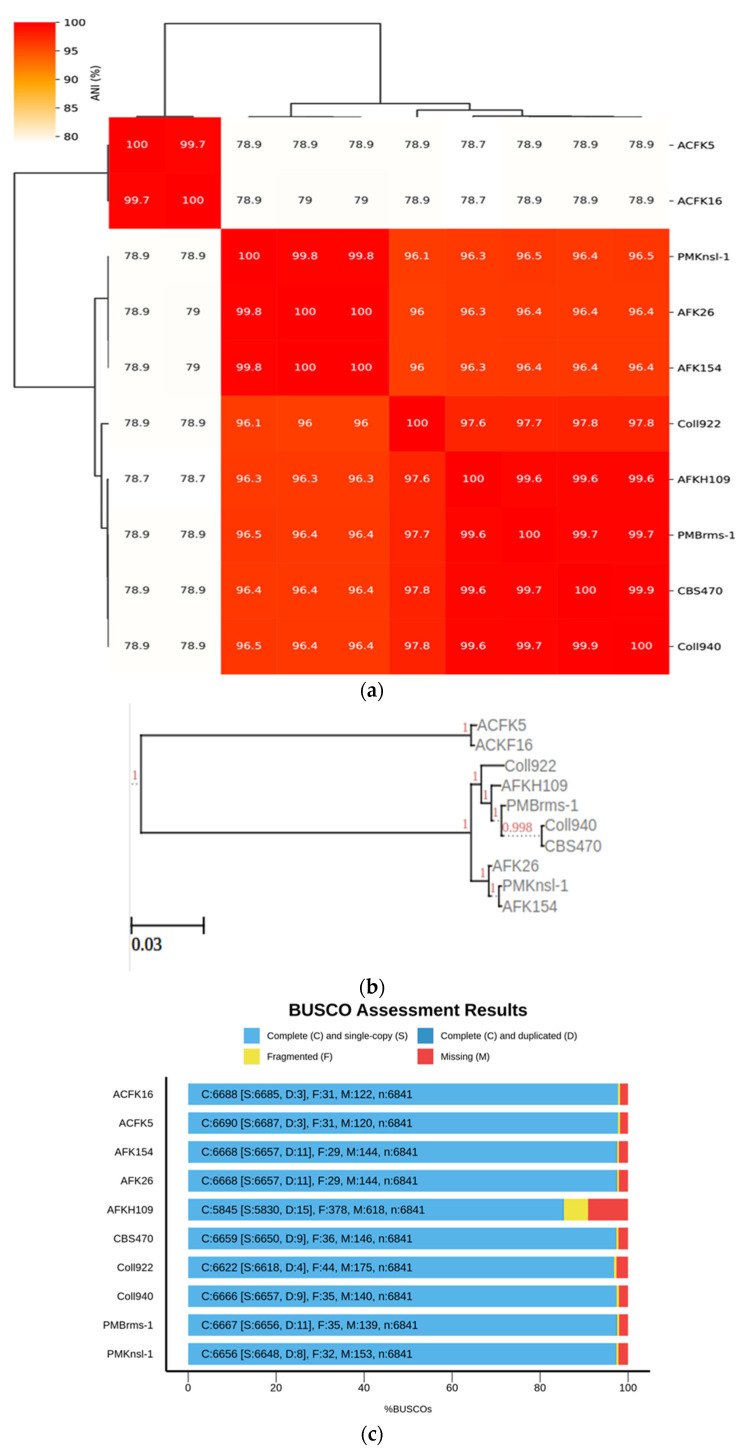

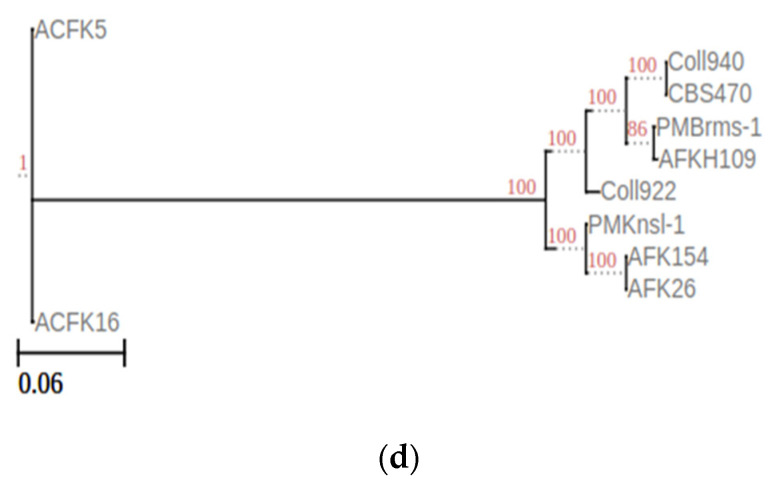
Phylogenomic analysis of *Colletotrichum* species using multiple approaches at the genome level. Constructing phylogenetic trees is based on the following: (**a**) High-throughput average nucleic identity (ANI) that documents genome sequence similarity. Numbers in the heat map represent percent similar identity. (**b**) Conserved gene sequence similarity as determined from Orthofinder [29]. Branch lengths and bootstrap support are shown. (**c**) BUSCO gene sets from the Glomerellales ODB10 database. (**d**) Highly conserved single-copy orthologous genes using BUSCO_phylogenomics pipeline. Branch lengths were optimized by maximum likelihood from the original alignment. Numbers on the branches represent the bootstrap support percentage.

The current study was expanded to encompass a larger dataset, incorporate the informative *APN2* locus known to enhance resolution within CGSC, and evaluate the novel clade using the stringent Genealogical Concordance Phylogenetic Species Recognition (GCPSR) criteria. This comprehensive approach confirmed the isolates as a strongly supported clade, distinct from all other taxa within CGSC [6]. These substantial morphological disparities between *C. noveboracense* and *C. nupharicola* warranted a more comprehensive analysis. To understand the evolutionary relationships among species, a genome-based method was utilized. The alignment-free method via fastANI software was used and is typically implemented in the determination of species for prokaryotes. However, previous works using small Eukaryote (fungal) genomes and genes were successful [41,42]. When employed for our dataset, ANI identified a group with a similar sequence identity that contained ACFK5 and ACFK16 belonging to CASC, whereas the remaining eight assemblies, belonging to CGSC, grouped together with ANI values of 96.5–99.9% similarity (Figure 1a). For our three assemblies in question, AFKH109, Coll940, and PMBrms-1 grouped together with CBS470 into subclades branching from Coll922. The same result was observed when constructing a tree based on gene trees of orthologs shared between assemblies (Figure 1b). This result was determined using ortholog trees based on protein similarities via the OrthoFinder software [29]. Another independent approach used BUSCO gene sets from the Glomerellales ODB10 database (*n* = 6841). Over 6618 single-copy BUSCO sequences were identified in each genome, except for AFKH109 which had 5830 (Figure 1c). This analysis suggests that the AFKH109 assembly is of lower quality. We used the BUSCO_phylogenomics pipeline to construct phylogenetics trees using these highly conserved gene sets (Figure 1d). The pipeline used 6714 BUSCO sequences that were complete, single-copy, and were found in at least four assemblies. The resulting consensus tree was highly similar to the prior two approaches (phylogenetic and sequence similarity) but separated ACFK5 and ACFK16 from a single clade. Again, AFKH109, Coll940, and PMBrms-1 grouped together with CBS470 into a subclade with the same subtending branch for Coll922. Consistently across all three methods, CBS470 and Coll940 were most closely related. AFKH109 and PMBrms-1 either grouped together or were subtending the CBS470 and Coll940 clades in that order, respectively. Based on our genome analysis, CBS470 (*C. nupharicola*) exhibits a closer genetic similarity to three *C. noveboracense* isolates (Coll940, PMBrms-1, and AFKH109) compared to other isolates. This suggests a potentially closer evolutionary relationship between *C. nupharicola* and these specific *C. noveboracense* isolates. As a complementary approach, we also reconstructed the BUSCO phylogeny and fastANI analysis using previously identified *acutatum* subclade members (Supplementary Figure S1A,B) [35]. These broader analyses support our previously stated conclusions as the phylogenetic structure was preserved between our nine subject genomes and the broader *Colletotrichum* species members (Supplementary Figure S1A). Moreover, the average nucleotide identity preserved these relations in this broader comparison (Supplementary Figure S1B). This finding supports previous phylogenetic efforts using eight informative loci [6]. The inferred phylogeny suggests a significant evolutionary distance between CASC and CGSC.

### 3.3. Bioinformatic Prediction of Effectors

Comparative genomics approaches lend themselves to the discovery of an array of predicted effectors, CAZymes, and SM gene clusters, which may not have otherwise been elucidated by classical genetic, pathological, and/or biochemical approaches. These studies often lead to the elucidation of both known and novel loci that can then be targeted for functional studies. Hence, we sought to utilize the *Colletotrichum* spp. genomes that we previously elucidated [27], with tools to discover a holistic view of factors that are routinely associated with fungal virulence, pathogenicity, and small molecule/toxin potential.

**Figure 2:**
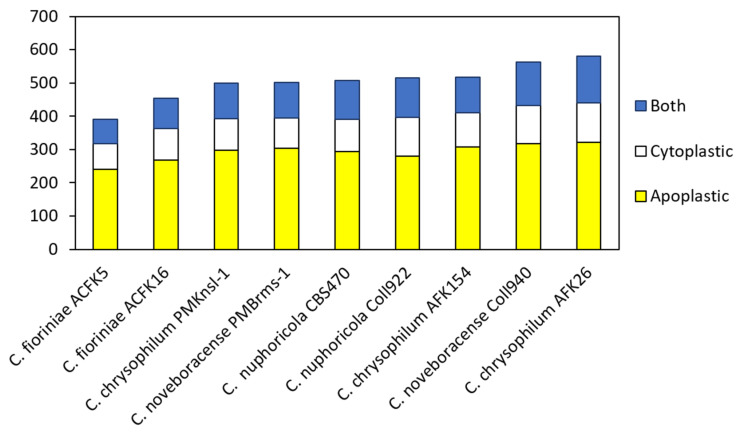
Distribution of predicted cytoplasmic, apoplastic, and dual-localized protein effectors in nine isolates of four *Colletotrichum* species.

Effector prediction was conducted using EffectorP 3.0 for nine different isolates consisting of four different *Colletotrichum* spp. [38,39]. This tool has been implemented to mine many different fungal genomes and has been recently used to explore *Colletotrichum truncatum* [22,43]. In our study, we found 390 to 581 predicted effectors with the lowest number in *C. fioriniae* and the highest number in *C. chrysophilum* AFK26 (Figure 2). Predicted apoplastic effectors ranged from 241 to 321 and cytoplasmically targeted effectors ranged from 77 to 120. Some effectors were categorized as both having the potential to be localized in either the cytoplasm or apoplasm. Rao and Nandeneni [22] found between 200 and 400 predicted effectors in four different *Colletotrichum* spp., of which *C. fioriniae* showed around 300 which is similar to our findings. Effector prediction using EffectorP 3.0 in Lu et al.’s [14] study identified a large number of candidate effectors in different *Colletotrichum* species (288 to 608 per genome). This analysis suggests significant variation in predicted effector numbers between *Colletotrichum* species. Further clustering analysis within the genus revealed that about 20% of these candidate effectors are core effectors, present in all *Colletotrichum* species examined. Another 70% represent conserved effectors with orthologs (similar genes) identified in some *Colletotrichum* species. Interestingly, each species also harbors a unique set of species-specific effectors, ranging from 4.1% to 15.6% of their total predicted effectors. These findings suggested a potential link between the conservation patterns of candidate effectors and the host range and virulence of *Colletotrichum* pathogens [14]. Moreover, de Queiroz et al. [44] conducted a comprehensive analysis of candidate effector proteins in two physiological races (83.501 and 89 A2 2-3) of the pathogen *Colletotrichum lindemuthianum* and found a total of 353 and 349 effectors, respectively. Interestingly, over 63% of these effectors share common features: they are rich in cysteine, contain repetitive amino acid sequences, and/or possess nuclear localization signals [44]. Additionally, analysis revealed several conserved protein domains shared among the *C. lindemuthianum* effector candidates. Later, they extended the analysis to nine other *Colletotrichum* species. The number of predicted effectors varied across these species, ranging from 247 in *Colletotrichum graminicola* to 446 in *Colletotrichum orbiculare*. Notably, all analyzed species shared twelve conserved protein domains within their predicted effector candidates [44]. Discrepancies in numbers determined in this study, with isolates from other studies, and between isolates of a similar species could be due to an array of factors that relate to genome coverage, annotation quality, and differences in bioinformatic programs utilized to analyze various genomes. It is of interest that overall, the total numbers of predicted effectors can be generalized as three *Colletotrichum* spp. (*C. nupharicola, C. noveboracense,* and *C. chrysophilum*) had higher numbers than *C. fioriniae.* Notwithstanding, the biological ramifications of these observations may not be realized at this time and require systematic functional study in the fungus via gene deletion, RNA silencing, and/or overexpression in planta to ascertain their role in fungal–host interactions.

### 3.4. Carbohydrate Active Enzyme (CAZyme) Classes

**Figure 3:**
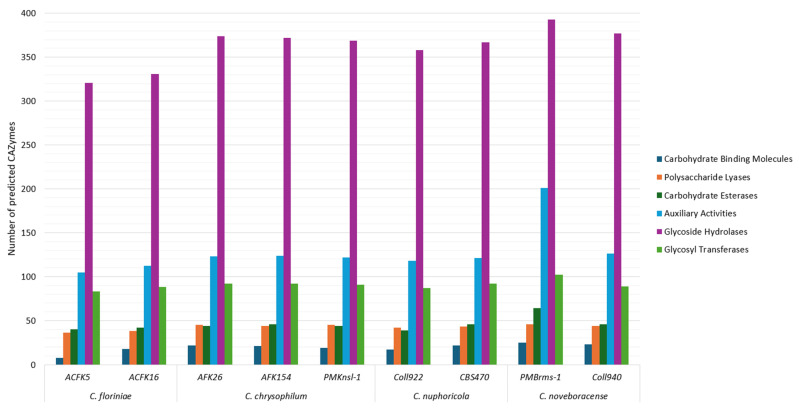
CAZyme prediction in nine sequenced isolates of four *Colletotrichum* species. Columns represent different CAZyme categories in the following order from left to right: carbohydrate-binding molecules, polysaccharide lyases, carbohydrate esterases, auxiliary activities, glycoside hydrolases, and glycosyl transferases.

CAZymes are enzymes involved in complex carbohydrate synthesis or breakdown [45]. These enzymes are enriched in many fungal taxa including those whose members engage in plant associations (e.g., phytopathogens). The study and identification of CAZymes has been of great interest due to their biotechnological potential and the possibility of using their profiles as clues to understand fungal lifestyles and their evolution which includes plant pathogenic potential [46,47]. This study showed the comparison of carbohydrate-active enzyme (AA, CE, GH, and PL) profiles between species, providing insights into their diverse carbohydrate degradation capabilities (Figure 3). A previous study showed that plant pathogenic fungi contain the largest number of CAZymes [47]. In this study, the total number of predicted CAzyme genes for the sequenced isolates ranged from 593 to 831. This is a high number when compared to other species in the genus like *C. australisiense* with 541 genes and *C. siamense* with 507 genes but similar when compared to *Colletotrichum camelliae* with 836 genes [48,49]. All the assessed isolates show a similar trend in the abundance of each identified CAZyme category (Figure 3). The identified categories from least to most abundant CAZymes in all the analyzed species are as follows: (1) carbohydrate-binding molecules (CBMs), (2) polysaccharide lyases (PLs), (3) carbohydrate esterases (CEs), (4) glycosyl transferases (GTs), (5) auxiliary activities (AAs), and (6) glycoside hydrolases (GH)s. While glycoside hydrolases are the CAZyme category with the most predicted genes for the assessed *Colletotrichum* spp. in this study, pectin, a glycoside hydrolase substrate, comprises from 5.6 to 10% of the apple cell wall components and hemicellulose 2 to 4.1% [50]. This bioinformatic prediction sheds light on the metabolic potential of these species, on understanding the basic biology of virulence, and the potential industrial application for the degradation of complex carbohydrates. In addition, gene expression analyses, for individual loci, specific CAZyme classes, and a systems-based RNAseq/functional approach of these genes in *Colletotrichum* spp. will leverage our genomics findings to further understand how these loci enhance host specificity and/or underpin aggressiveness in host–parasite interactions.

### 3.5. Biosynthetic Gene Cluster Distribution

**Figure 4:**
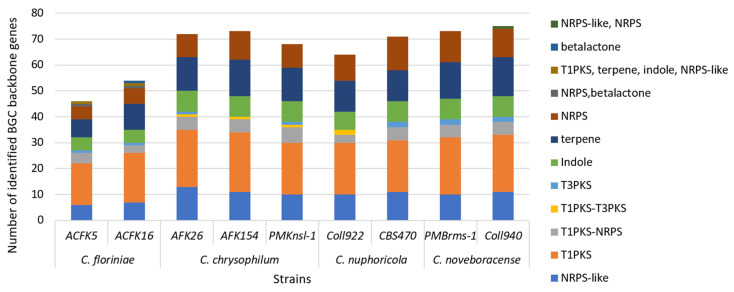
SM backbone gene cluster prediction in nine isolates of four *Colletotrichum* species. Columns represent the total number of identified backbone genes per isolate after using antiSMASH 6.0 pipeline. Each column contains the identified categories and their frequency per isolate.

Fungi produce unique molecules (natural products/secondary metabolites) through specialized biosynthetic gene clusters (BGCs). Unlike those for growth, these BGCs (2–20+ genes) create a chemical arsenal (toxins and antibiotics) vital for fungal interactions with other microbes and their environment [51]. In this study, we used antiSMASH to predict the nine *Colletotrichum* spp. SM gene cluster repertoire. We found that the number of predicted SM gene clusters ranged from 46 in *C. floriniae* ACFK5 to 75 in *C. noveboracense* Coll940 (Figure 4). These numbers of clusters are typical for the genus as previous studies have reported 71 for *C. camelliae*, 85 SM gene clusters for *Colletotricum siamense,* and 55 for *Colletotricum australisinense* [48,49]. In addition, all of the sequenced strains contained similar profiles in the type of BGC that were predicted. The assessed species contain the greatest proportion of type-1 polyketide synthases, i.e., T1PKS-type backbone genes in their genomes, followed by terpene backbone genes, and finally nonribosomal peptide-synthetase (NRPS) or NRPS-like genes. These findings are congruent with what Liu et al. [48] found when comparing *C. siamense* and *C. australisinense*. The cercosporin gene cluster is present for all the assessed samples but its homology varies between the sequenced isolates to the representative cluster of *Cercospora betiicola* [52]. The role of polyketide synthases (PKSs) in promoting host penetration by *Colletotrichum* species has been recognized for a considerable time [53]. While all the SM gene cluster predictions are valuable, most of the detected clusters have not been characterized, representing a great opportunity to further mine, characterize, and understand their ecological role in *Colletotrichum* spp. Secondary metabolites (SMs) produced by fungal phytopathogens exhibited a robust correlation with both their pathogenicity and host range [22]. Application of these phytotoxic SMs to host leaves induced disease symptoms mirroring those observed in anthracnose caused by *Colletotrichum* species. This finding underscores the critical role these metabolites play in the pathogenesis and infection mechanisms employed by these fungi [54].

Of the reported BGCs in this study, alternapyrone is conserved throughout all the assessed *Colletotrichum* species. Alternapyrone is a polyketide predicted to be synthesized by five gene products named altA to altD [55]. For this cluster, the backbone gene encodes for an iterative type I polyketide synthase (pksN), one gene encodes for a FAD/FMN-dependent oxygenase/oxidase, and three genes are annotated as a cytochrome P-450. The alternapyrone BGC was first described in *Alternaria solani*, the early blight disease pathogen of tomato and potato, and recently has been reported to be produced by *Parastagonospora nodorum*, a fungal wheat pathogen [55,56]. Limited information can be found on the compound bioactivity, but data suggest that alternapyrone displays cytotoxic activity and some derivatives inhibit wheat seed germination [56]. This finding along the complete BGC could further be explored to unravel individual species secondary metabolite production and the importance of their role in ecological and interspecies interactions. This research holds significant promise for the improved management of apple bitter rot. Our work provides a foundation for developing targeted control strategies by identifying candidate genes crucial for *Colletotrichum* spp. virulence and pathogenicity. These genes, including effectors, CAZyme genes, and secondary metabolite gene clusters, represent potential targets for novel control mechanisms. RNA interference or gene editing techniques could be employed to silence or disrupt these genes, hindering fungal function. Furthermore, by comparing the genomes of various *Colletotrichum* species that infect apples, this study sheds light on the genetic basis of host specificity. This newfound knowledge can be harnessed to develop apple cultivars exhibiting enhanced resistance to bitter rot disease.

## 4. Conclusions

This study utilized whole genome sequencing of nine *Colletotrichum* isolates encompassing four species (*C. fioriniae*, *C. chrysophilum*, *C. noveboracense,* and *C. nupharicola*) to gain insights into their pathogenicity, host specificity, and evolutionary relationships. Phylogenomic analyses confirmed the distinctiveness of *C. noveboracense* as a novel causal agent of apple bitter rot disease. Notably, *C. noveboracense* and *C. nupharicola* displayed a closer evolutionary relationship compared to other species in the CGSC and the CASC. Comparative genomics revealed a vast repertoire of potential virulence factors, including predicted effector proteins, carbohydrate-active enzymes (CAZymes), and secondary metabolite (SM) gene clusters. To solidify our findings and translate them into actionable strategies, future research should pursue several avenues. Functional validation of the identified candidate genes is crucial. Techniques like targeted gene deletion, overexpression, and RNA silencing can elucidate which genes are essential for *Colletotrichum* spp. virulence and pathogenicity. Additionally, omics approaches such as transcriptomics, proteomics, and metabolomics offer a deeper understanding of gene expression during infection and the production of metabolites by *Colletotrichum* spp. These comprehensive data can reveal further targets for control strategies. Finally, the wealth of genetic information gleaned from this study holds immense potential for developing more specific and accurate molecular diagnostic tools [57]. These tools can effectively identify *Colletotrichum* spp. responsible for apple bitter rot, facilitating earlier intervention and improved disease management. Our findings provide a valuable resource for further functional studies aimed at elucidating the specific roles of these virulence factors in *Colletotrichum* spp. Additionally, these data lay the groundwork for the development of novel and targeted disease management strategies for crops susceptible to *Colletotrichum* infection.

## Figures and Tables

**Table 1:** Summary of draft genome assembly and annotation metrics for *Colletotrichum* species (Khodadadi et al. [27]).

*Colletotrichum* Species	Isolates	Genome Accessions Numbers	Genome Size (Mb)	Contigs	N_50_ Contig Length	Number of Genes
*C. fioriniae*	ACFK16	GCA_026319165.1	49.33	514	742,368	12,377
	ACFK5	GCA_026319145.1	49.44	169	688,115	11,886
*C. nupharicola*	CBS470	GCA_026319135.1	58.75	3696	137,264	13,425
	Coll922	GCA_026319225.1	51.67	39,421	34,056	14,546
*C. chrysophilum*	AFK154	GCA_026319245.1	57.85	1702	268,763	13,999
	AFK26	GCA_026319265.1	55.97	1029	288,101	14,123
	PMKnsl-1	GCA_026319215.1	56.06	1954	255,107	13,409
*C. noveboracense*	Coll940	GCA_026319155.1	58.17	1007	168,995	14,011
	PMBrms-1	GCA_026319125.1	59.08	2959	176,901	13,397

## Data Availability

Data are contained within the article, Supplementary Materials, and genome sequences used in this research are deposited in NCBI’s GenBank (you can find their accession numbers in Table 1).

## Supplementary Materials

The following supporting information can be downloaded at https://www.mdpi.com/article/10.3390/jof10070493/s1; Table S1: Summary of *Colletotrichum* spp. reference genomes from the *acutatum* subclade used in the expanded phylogenic analyses; Figure S1: Phylogenomic analysis of *Colletotrichum* species within the acutatum clade using multiple approaches at the genome level. Constructing phylogenetic trees based on: A. High-throughput average nucleic identity (ANI) that documents genome sequence similarity. Numbers in the heat map represent percent similar identity.; B. Highly conserved single copy orthologous genes using BUSCO_phylogenomics pipeline. Branch lengths were optimized by maximum likelihood from the original alignment. Numbers on the branches represent the bootstrap support percentage.
