# Supplementary material for: Unveiling the Arsenal of Apple Bitter Rot Fungi: Comparative Genomics Identifies Candidate Effectors, CAZymes, and Biosynthetic Gene Clusters in Colletotrichum Species
Source: J Fungi (Basel). 2024 Jul 16;10(7):493. doi: 10.3390/jof10070493 (PMC11278308; doi:10.3390/jof10070493)
Supplement: Supplementary file 1 [file jof-10-00493-s001.zip › Supplemental Figure S1.pptx]

## Slide 1
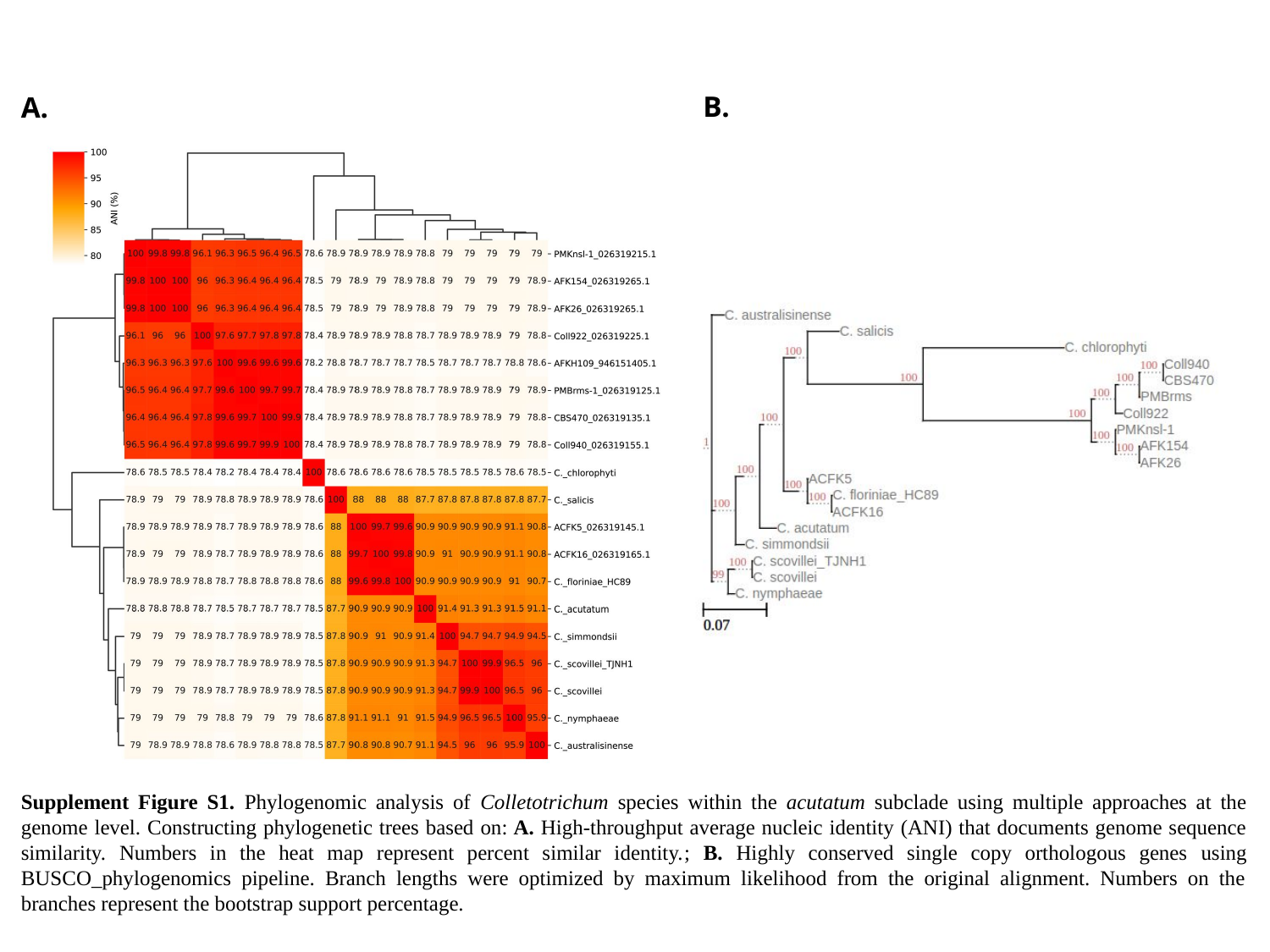

B.
A.
Supplement Figure S1. Phylogenomic analysis of Colletotrichum species within the acutatum subclade using multiple approaches at the genome level. Constructing phylogenetic trees based on: A. High-throughput average nucleic identity (ANI) that documents genome sequence similarity. Numbers in the heat map represent percent similar identity.; B. Highly conserved single copy orthologous genes using BUSCO_phylogenomics pipeline. Branch lengths were optimized by maximum likelihood from the original alignment. Numbers on the branches represent the bootstrap support percentage.
